# Pharmacological Characterization of Orofacial Nociception in Female Rats Following Nitroglycerin Administration

**DOI:** 10.3389/fphar.2020.527495

**Published:** 2020-12-03

**Authors:** Robert M. Caudle, Stephanie L. Caudle, Natalie D. Flenor, Eric L. Rohrs, John K. Neubert

**Affiliations:** ^1^ Department of Oral and Maxillofacial Surgery, University of Florida, Gainesville, FL, United States; ^2^ Department of Orthodontics, University of Florida, Gainesville, FL, United States; ^3^ Velocity Laboratories, LLC, Alachua, FL, United States

**Keywords:** nitroglycerin, orofacial pain, operant assay, migraine, rodent, female

## Abstract

Rodent models of human disease can be valuable for understanding the mechanisms of a disease and for identifying novel therapies. However, it is critical that these models be vetted prior to committing resources to developing novel therapeutics. Failure to confirm the model can lead to significant losses in time and resources. One model used for migraine headache is to administer nitroglycerin to rodents. Nitroglycerin is known to produce migraine-like pain in humans and is presumed to do the same in rodents. It is not known, however, if the mechanism for nitroglycerin headaches involves the same pathological processes as migraine. In the absence of known mechanisms, it becomes imperative that the model not only translates into successful clinical trials but also successfully reverse translates by demonstrating efficacy of current therapeutics. In this study female rats were given nitroglycerin and nociception was evaluated in OPADs. Estrous was not monitored. Based on the ED_50_ of nitroglycerin a dose of 10 mg/kg was used for experiments. Sumatriptan, caffeine, buprenorphine and morphine were administered to evaluate the reverse translatability of the model. We found that nitroglycerin did not produce mechanical allodynia in the face of the rats, which is reported to be a consequence of migraine in humans. Nitroglycerin reduced the animals’ participation in the assay. The reduced activity was verified using an assay to measure exploratory behavior. Furthermore, the effects of nitroglycerin were not reversed or prevented by agents that are effective acute therapies for migraine. Two interesting findings from this study, however, were that morphine and nitroglycerin interact to increase the rats’ tolerance of mechanical stimuli on their faces, and they work in concert to slow down the central motor pattern generator for licking on the reward bottle. These interactions suggest that nitroglycerin generated nitric oxide and mu opioid receptors interact with the same neuronal circuits in an additive manner. The interaction of nitroglycerin and morphine on sensory and motor circuits deserves additional examination. In conclusion, based on the results of this study the use of nitroglycerin at these doses in naïve female rats is not recommended as a model for migraine headaches.

## Introduction

Migraine headaches are one of the most common debilitating chronic pain conditions, affecting more than ten percent of the global population (Woldeamanuel and Cowan, 2017). Triptans, opioids, NSAIDs and caffeine are commonly used to abort migraine headaches and are relatively effective, but prevention of migraines remains problematic. Recently, botulinum toxin, calcitonin gene-related peptide (CGRP) antagonists and antibodies to CGRP and CGRP receptors have been developed for long term control of migraine headaches (Matak et al., 2011; Dodick et al., 2014a; Dodick et al., 2014b; Lupi et al., 2019). However, these therapies typically reduce the incidence of migraine by just a few attacks per month (Dodick et al., 2019; Urits et al., 2019), indicating that extended therapy for chronic migraine is still not satisfactory. Given the debilitating nature of migraine headaches and the large number of people who suffer from the disease novel therapies are needed.

One common model for migraine headache drug discovery programs is to give rodents a nitric oxide donor such as nitroglycerin to produce headaches. Nitroglycerin induces the activation of guanylate cyclase, stimulating cGMP formation, which in turn leads to a vasodilatory effect (Seiler et al., 2013). Nitroglycerin is usually prescribed for angina patients (Bray et al., 1991), but headaches are a frequent side effect (Schwartz, 1946). This side effect led to nitroglycerin being used to model migraine headaches. However, because the pathologic mechanisms for migraine headache are not well established and nitroglycerin produces global pain in the head rather than focal pain like migraines it is not clear that nitroglycerin is truly modeling the disease. Furthermore, in a recent study triptans did not reverse nitroglycerin induced headaches in humans even though they are a highly effective abortive therapy for the majority of migraine sufferers (Tvedskov et al., 2010). A previous study by the same researchers, however, did indicated some degree of headache prevention when sumatriptan was given prior to nitroglycerin in healthy volunteers (Iversen and Olesen, 1996). These observations suggest that nitroglycerin in humans may not be an accurate model of migraine headache.

Despite the differences between nitroglycerin induced headache and migraine headache a significant number of migraine studies have utilized rodent nitroglycerin models. These studies often demonstrate that triptans reverse nitroglycerin induced hypersensitivity in rodents using reflex based thermal and mechanical sensitivity on the limbs or tail rather than examining sensitivity in trigeminal nerve territories, which would be more appropriate for migraine (Tassorelli et al., 2003; Bates et al., 2010; Tipton et al., 2016; Moye et al., 2019). Other studies utilized von Frey mechanical assays in the periorbital region of rodents (Di et al., 2015; Kopruszinski et al., 2017; Marone et al., 2018). These experiments rely on the suppression of a pain induced enhancement of a behavioral response, such as a shortened paw or head withdrawal latency from a heat or mechanical stimulus. The animal’s response to the stimulus must be interpreted by an observer leading to the possibility that any sedation or change in motor function is interpreted as an anti-nociceptive response in these models. Furthermore, it is difficult to blind the investigators running the assay to the rodents’ treatment due to several characteristic behaviors displayed by the rodents when they are treated with nitroglycerin, e.g., eye squinting, stretching, and reduced motor activity. These behaviors may bias the investigator during the testing process and have little to do with the desired behavioral outcome. Another issue with the use of investigator evoked responses in rodents is that nitroglycerin may produce a state of hypervigilance in which the animals respond to the detection of the stimulus rather than responding only to nociceptive stimuli. The hypervigilance may lead to the misinterpretation of the rodent’s experience with the stimulus and any agent that reduces the animal’s anxiety may appear to be analgesic in these assays.

To address the adequacy of nitroglycerin in rats as an acute model of human migraine headache this study evaluated the model pharmacologically in an operant assay using Orofacial Pain Assessment Devices (OPAD, Stoelting, Co.). The hypothesis tested was that agents that can abort human migraine headaches would disrupt nitroglycerin induced headaches in rats. The advantages of the OPAD testing system are that 1) it assesses nociception in trigeminal nerve territories, which is more relevant to migraine than limb sensitivity; 2) data collection is automated so that investigator bias is reduced; 3) the assay utilizes a behavior that is suppressed by pain so that effective analgesics restore the behavior; thus, sedation or motor impairment do not register as analgesic effects; 4) the assay utilizes a rodent initiated behavior rather than an investigator evoked behavior which reduces the impact of rodent hypervigilance; and 5) the assay is an operant reward/conflict type of assay that quantifies the full experience of the pain including the nociceptive, and cognitive/emotional elements of the experience rather than measuring a simple reflex arc (Neubert et al., 2005; Neubert et al., 2006; Rossi et al., 2006; Neubert et al., 2007; Neubert et al., 2008; Rossi and Neubert, 2008; Rossi et al., 2009; Caudle et al., 2010; Kumada et al., 2012; Nolan et al., 2011; Nolan et al., 2012; Ramirez and Neubert, 2012; Rossi et al., 2012; Anderson et al., 2013; Mustafa et al., 2013; Anderson et al., 2014; Ramirez et al., 2015; Bowden et al., 2017; Caudle et al., 2017; Sapio et al., 2018). By evaluating agents that are effective in the acute clinical management of migraine headache in humans in this rat nitroglycerin model the validity of the model was tested. The results of this reverse translation study indicate that the treatment of rats with nitroglycerin is not likely to be a valid model of human migraine headache.

## Materials and Methods

### Animals

Female Sprague Dawley rats (200–350 g, Charles Rivers) were utilized for the experiments. The animals were fed standard rodent chow and water ad libitum throughout the study and were housed in pairs at 22°C with 30% humidity. The rooms were on a 12-h light/dark cycle (7 AM–7 PM lights on). The animals were not food or water restricted prior to training or testing and estrus cycles were not monitored. All experiments were carried out in accordance with the Guide for the Care and Use of Laboratory Animals and were reviewed and approved by the University of Florida Institutional Animal Care and Use Committee.

### Orofacial Pain Assessment Device Behavioral Assay

**FIGURE 1:**
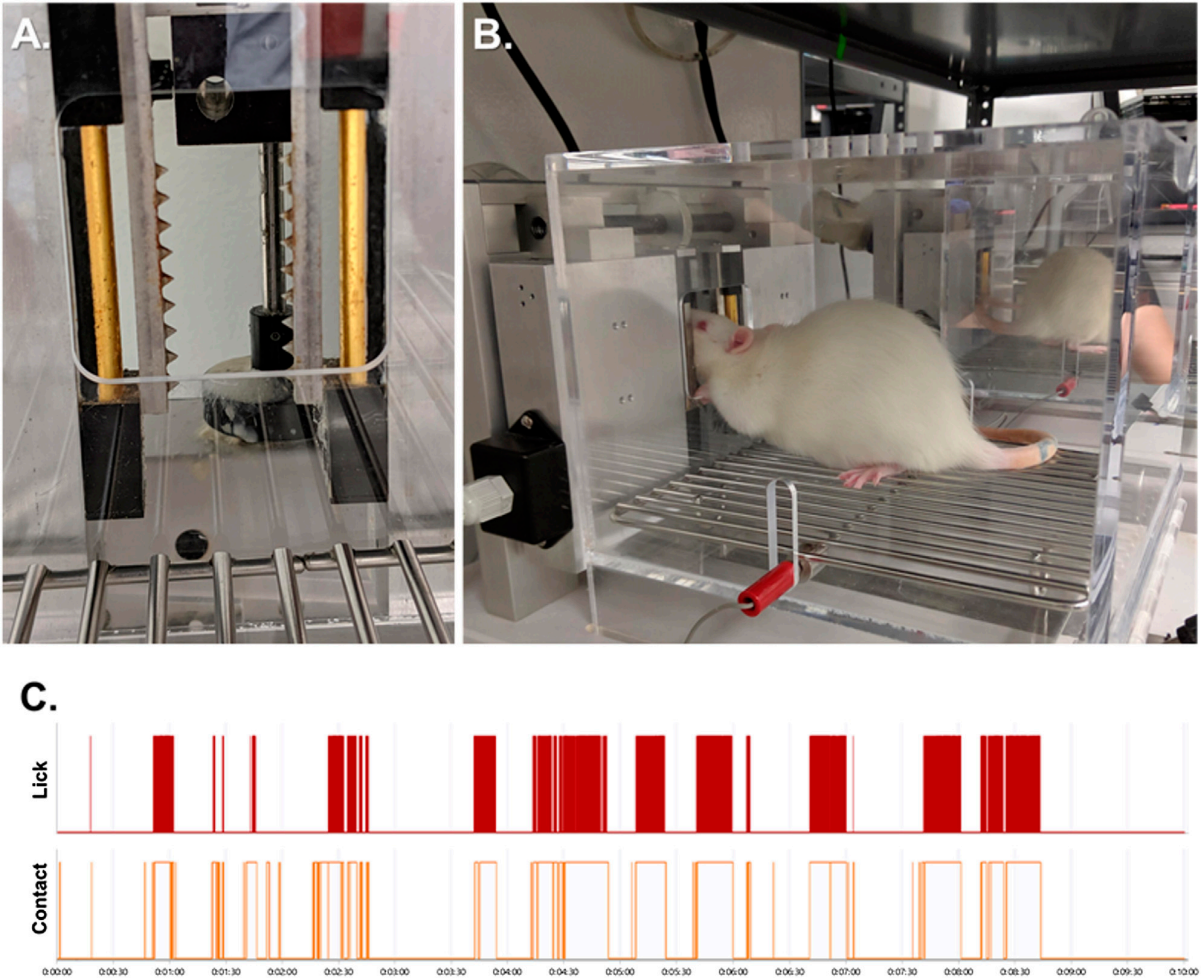
The Orofacial Pain Assessment Device (OPAD) for Mechanical Nociception. **(A)** Spiked bars that the rats must press their faces against to obtain a reward solution. **(B)** Example of a rat performing the OPAD assay. **(C)** Examples of raw licking and stimulus contact data collected in the OPAD during a 10-min testing session.

Standard OPADs (Stoelting Co.) were fitted with spiked bars on each side of the reward solution access window (Figure 1A). The spikes on the bars were spaced 5 mm apart and extended 3 mm into the opening. The bars were positioned relative to the reward solution bottle so that to obtain the reward the rats had to press their faces firmly against the spikes (Figure 1B). The reward solution was sweetened condensed milk diluted with water (1 milk: 2 water) and the stimulus bars were kept at room temperature (∼22–23°C).

Rats were trained daily in the OPADs between the hours of 10:00 to 12:00 Monday through Friday. Ten training sessions were completed prior to beginning experiments. Experiments were also conducted between 10:00 and 12:00 and each session was 10 min in duration. The rodents’ licks on the reward bottle and their contact with the stimulus bars were recorded electronically using AnyMaze software (Stoelting Co.). An example of the data collected is presented in Figure 1C. The use of the OPAD assay has been described extensively in our previous publications (Neubert et al., 2005; Neubert et al., 2006; Neubert et al., 2007; Neubert et al., 2008; Rossi and Neubert, 2008; Rossi et al., 2009; Caudle et al., 2010; Kumada et al., 2012; Nolan et al., 2011; Anderson et al., 2012a; Anderson et al., 2012b; Nolan et al., 2012; Ramirez and Neubert, 2012; Rossi et al., 2012; Anderson et al., 2013; Mustafa et al., 2013; Anderson et al., 2014; Anderson et al., 2015; Ramirez et al., 2015; Bowden et al., 2017; Caudle et al., 2017).

### Rearing Activity Assay

Our previously described rearing test was used as an assessment of motor activity and exploratory behavior (Neubert et al., 2007; Rossi and Neubert, 2008). Briefly, an acrylic cylinder (19.5 cm diameter × 40.5 cm height) was constructed with aluminum sheets placed both on the floor and 13.5-cm above the floor. The metal siding was connected to a DC power supply (12 V) and, in series, to a multi-channel data acquisition module (DATAQ Instruments, Inc.). The floor of the cylinder served as the ground for the circuit. Unrestrained animals were placed into separate cylinders and the data acquisition software (WinDaq, DATAQ Instruments, Inc.) was activated. When an animal reared its front paws would contact the metal side of the cylinder completing an electrical circuit with the grounded floor. The closed circuit was registered on the computer. Each session was 12.75 min in duration.

### Drug Treatments

Nitroglycerin (7.5, 10, or 15 mg/kg, i.p., Henry Schein) was administered 20 min prior to the initiation of a 10-min OPAD session for acute experiments. For the repeated exposure experiments nitroglycerin (10 mg/kg, i.p.) was administered 30 min after OPAD testing for five consecutive days.

Morphine sulphate (1.5 and 10 mg/kg, Patterson Veterinary Supply, Inc.), buprenorphine (0.03 mg/kg, Patterson Veterinary Supply, Inc.), and caffeine (2 mg/kg dissolved in PBS, Sigma-Aldrich) were administered i.p. 30 min prior to testing in the OPADs. Sumatriptan succinate (0.3 mg/kg dissolved in PBS, Sigma-Aldrich) was administered i.p. 10 min prior to testing in the OPADs as a rescue treatment and in a separate group of rats 1 mg/kg sumatriptan succinate was administered i.m. 30 min prior to testing. Phosphate buffered saline (PBS, pH 7.4) was used as a vehicle and injection control. Naïve rats received no injections.

### Statistics

The licks on the reward bottle and the contacts with the stimulus bars were collected automatically with Stoelting’s AnyMaze software. To evaluate the interval between licks 20 consecutive intervals were manually sampled from a single bout of licking for each animal. The data analysis feature of AnyMaze was used to measure the interval from the offset of a lick to the initiation of the following lick.

Data was exported from AnyMaze to Excel and PRISM version 6.07 (GraphPad Software, Inc.) for statistical analyses. Non-linear regressions were used to fit dose response curves and determine ED50s. T-tests, One-way ANOVAs followed by Dunnett’s or Holm–Sidak’s multiple comparisons tests were used when appropriate. Data are presented as Mean ± SEM. Alpha was set to *p* ≤ 0.05.

## Results

### Behavioral Characterization of Nitroglycerin

**FIGURE 2:**
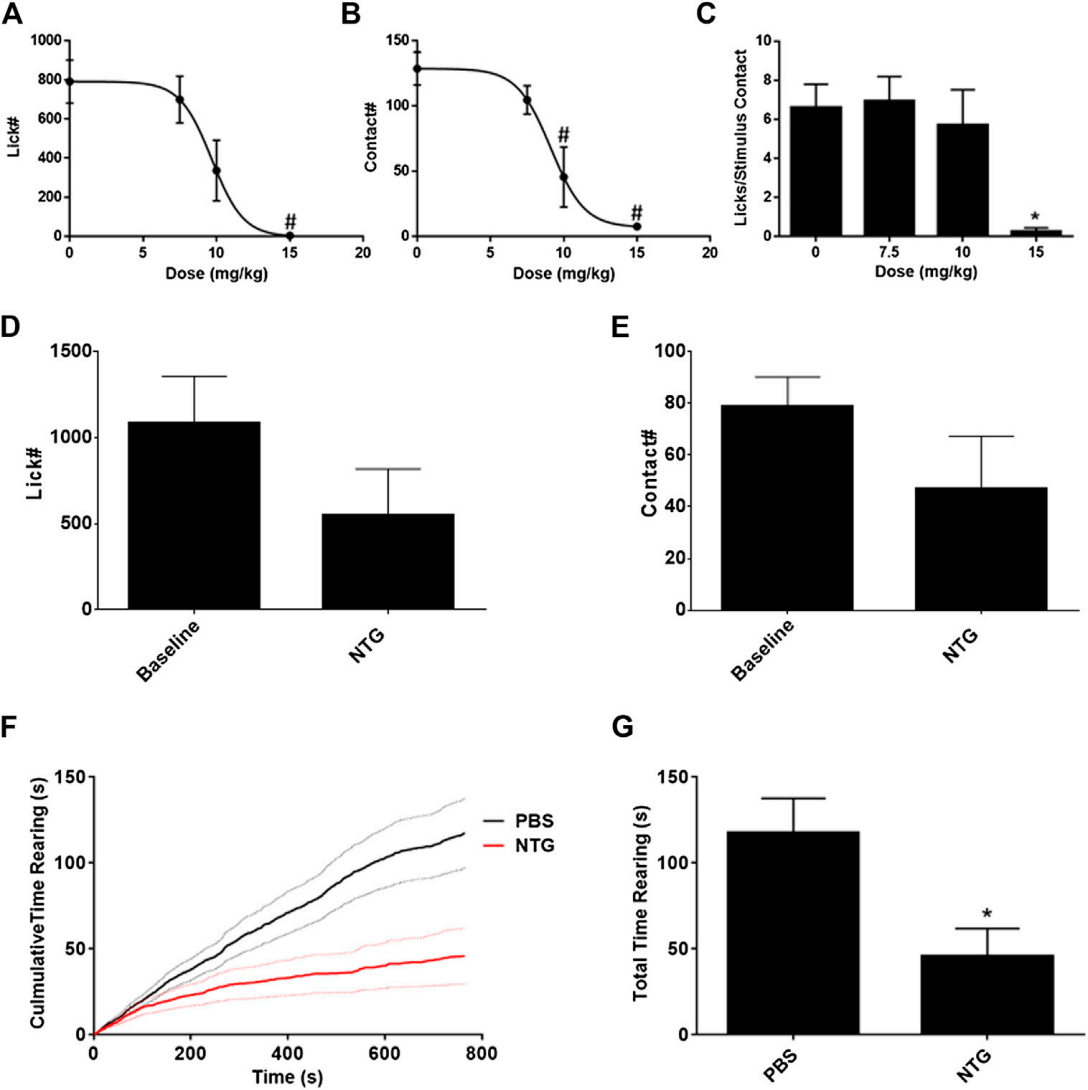
Effect of nitroglycerin on rat behavior. **(A)** Nitroglycerin dose response relationship on reward bottle licking in the OPAD assay. ED50 = 9.68 ± 1.00 mg/kg, One-way ANOVA: F (3, 56) = 6.79, *p* = 0.0006. #*p* < 0.05 Dunnett’s test when compared to 0 mg/kg. **(B)** Nitroglycerin dose response relationship on stimulus contacts in the OPAD assay. ED50 = 9.11 ± 0.85 mg/kg, One-way ANOVA: F (3, 56) = 12.99, *p* < 0.0001. #*p* < 0.05 Dunnett’s test when compared to 0 mg/kg. **(C)** Nitroglycerin dose response relationship on the ratio of lick to stimulus contacts in the OPAD assay. One-way ANOVA: F (3, 54) = 3.490, *p* = 0.0217. *p < 0.05 Holm-Sidak's multiple comparisons test when compared to 0 mg/kg. **(D)** Effect of nitroglycerin (10 mg/kg, i.p.) on licking in the OPAD assay 4 h following injection. (*N* = 7, Baseline vs NTG paired *t*-test: *t* = 1.812 df = 6, *p* = 0.120). **(E)** Effect of nitroglycerin on stimulus contacts in the OPAD assay 4 h following injection. (*N* = 7, Baseline vs. NTG paired *t*-test: *t* = 1.580 d*f* = 6, *p* = 0.165). **(F)** Effect of nitroglycerin (10 mg/kg, i.p.) in the rearing behavioral assay (*N* = 14 nitroglycerin, *N* = 15 PBS). Dashed lines are standard errors for the curves. **(G)** Nitroglycerin reduces total time rearing. * *t*-test: t = 2.758 df = 27, *p* = 0.010.

Nitroglycerin presumably produces a headache in rats like that experienced by humans. To determine an appropriate dose for testing against acute migraine headache therapies a dose response relationship was performed. As demonstrated in Figures 2A,B the ED50s for suppression of licks on the reward bottle and suppression of contacts with the stimulus bars were 9.68 ± 1.00 mg/kg and 9.11 ± 0.85 mg/kg respectively. The ratio of the licks to the stimulus contacts, which is utilized as an index of hypersensitivity (Neubert et al., 2005; Neubert et al., 2006; Neubert et al., 2007; Neubert et al., 2008; Caudle et al., 2010; Nolan et al., 2011; Ramirez and Neubert, 2012; Rossi et al., 2012; Mustafa et al., 2013; Ramirez et al., 2015; Sapio et al., 2018), was not altered until the highest dose of 15 mg/kg (Figure 2C). Because the total number of licks and the total number of stimulus contacts were very low at 15 mg/kg, and an ED50 could not be calculated from the curve, the suppression of the lick to stimulus contact ratio was not considered an indication of mechanical hypersensitivity. Instead, the data indicate that nitroglycerin reduced overall activity in the animals rather than producing mechanical hypersensitivity. This lack of hypersensitivity contrasts with previous reports on nitroglycerin in rodents (Di et al., 2016; Tipton et al., 2016; Farajdokht et al., 2018; Qin et al., 2018).

Based on the dose response data the dose of 10 mg/kg was utilized for subsequent studies, which is consistent with the work of others (Costa et al., 2005; Bates et al., 2010; Bobade et al., 2015; Farajdokht et al., 2018; Lai et al., 2019). The timing of the nitroglycerin administration relative to OPAD testing (20 min) was determined in preliminary studies based on the peak effect of nitroglycerin in the assay. Previous studies using various assays indicated that nitroglycerin induced hypersensitivity at time points as late as 4 h following injection (Guo et al., 2017; Lai et al., 2019). Figures 2D,E demonstrate that overall performance in the OPAD assay was slightly reduced at 4 h following 10 mg/kg nitroglycerin, but this effect did not reach statistical significance.

Because the OPAD data indicated that the rats’ total activity was suppressed by the nitroglycerin treatment a rearing assay was used to evaluate the motor and exploratory behavior of the rats when treated with nitroglycerin. The nitroglycerin was administered 20 min prior to testing. As indicated in Figures 2F,G, 10 mg/kg nitroglycerin significantly suppressed motor and exploratory activity in the rearing assay indicating that the effects of nitroglycerin disrupted their general activity levels, which may have contributed to the rats’ not seeking the reward solution in the OPAD assay.

### Effects of Therapeutic Agents in the Nitroglycerin Model

Two doses of sumatriptan that were demonstrated to be anti-nociceptive in previous rodent studies were tested against nitroglycerin (Tomić et al., 2015; Farkas et al., 2016). The low dose of sumatriptan (0.3 mg/kg, i.p.) was given as a rescue agent 10 min following the nitroglycerin injections and the high dose of sumatriptan (1 mg/kg, i.m.) was given as a preventative therapy. As demonstrated in Table 1 neither dose significantly influenced the effect of the nitroglycerin in the OPAD assay. Similarly, doses of morphine and caffeine that were previously shown to be effective anti-migraine agents and the mixed opioid agonist/antagonist buprenorphine did not inhibit nitroglycerin’s effects on the number of licks on the reward bottle or the number of contacts with the mechanical stimulus (Table 1) (Caudle and Isaac, 1987; Brasseur, 1997; Neubert et al., 2005; Neubert et al., 2006; Neubert et al., 2007; Neubert et al., 2008; Lobmaier et al., 2010; Nolan et al., 2012; Bird and Lambert, 2015; Ramirez et al., 2015; Tomić et al., 2015; Lipton et al., 2017; Walsh and Babalonis, 2017). The four agents also did not significantly alter the number of licks or stimulus contacts in the OPAD in the absence of nitroglycerin, indicating that they did not produce sedative effects that could have masked their antinociceptive actions.

**TABLE 1:** Effect of anti-nociceptive agents on nociception in the OPAD assay.

Treatment	*N*	Licks	Contacts
		Mean ± SEM	Mean ± SEM
Nitroglycerin (10 mg/kg)			
PBS	65	340.8 ± 55.7	45.0 ± 7.8
Sumatriptan 0.3 mg/kg	10	350.0 ± 136.2	51.0 ± 11.4
Sumatriptan 1.0 mg/kg	10	159.3 ± 81.6	18.5 ± 6.7
Caffeine	10	356.5 ± 134.4	62.9 ± 18.5
Buprenorphine	10	166.8 ± 89.4	10.5 ± 4.4
Morphine 1.5 mg/kg	10	506.5 ± 136.8	36.5 ± 10.4
Morphine 10 mg/kg	10	203.6 ± 160.7	9.8 ± 3.3
No Nitroglycerin			
Naïve	60	768.9 ± 78.9	112.4 ± 12.3
PBS	50	838.3 ± 101.5	127.3 ± 21.2
Sumatriptan 0.3 mg/kg	10	630.2 ± 225.8	58.6 ± 20.9
Caffeine	10	418.4 ± 159.8	194.2 ± 71.0
Buprenorphine	10	650.7 ± 265.5	105.8 ± 42.1
Morphine 1.5 mg/kg	10	826.3 ± 168.7	95.3 ± 23.0
Morphine 10 mg/kg	10	627.7 ± 228.4	42.4 ± 11.1

Nitroglycerin: One-way ANOVA Licks: F (6, 118) = 0.94, *p* = 0.47; One-way ANOVA Stimulus Contacts: F (6, 118) = 1.92, *p* = 0.08.

No Nitroglycerin: One-way ANOVA Licks: F (7, 162) = 0.90, *p* = 0.51; One-way ANOVA Stimulus Contacts: F (7, 162) = 1.66, *p* = 0.12.

**FIGURE 3:**
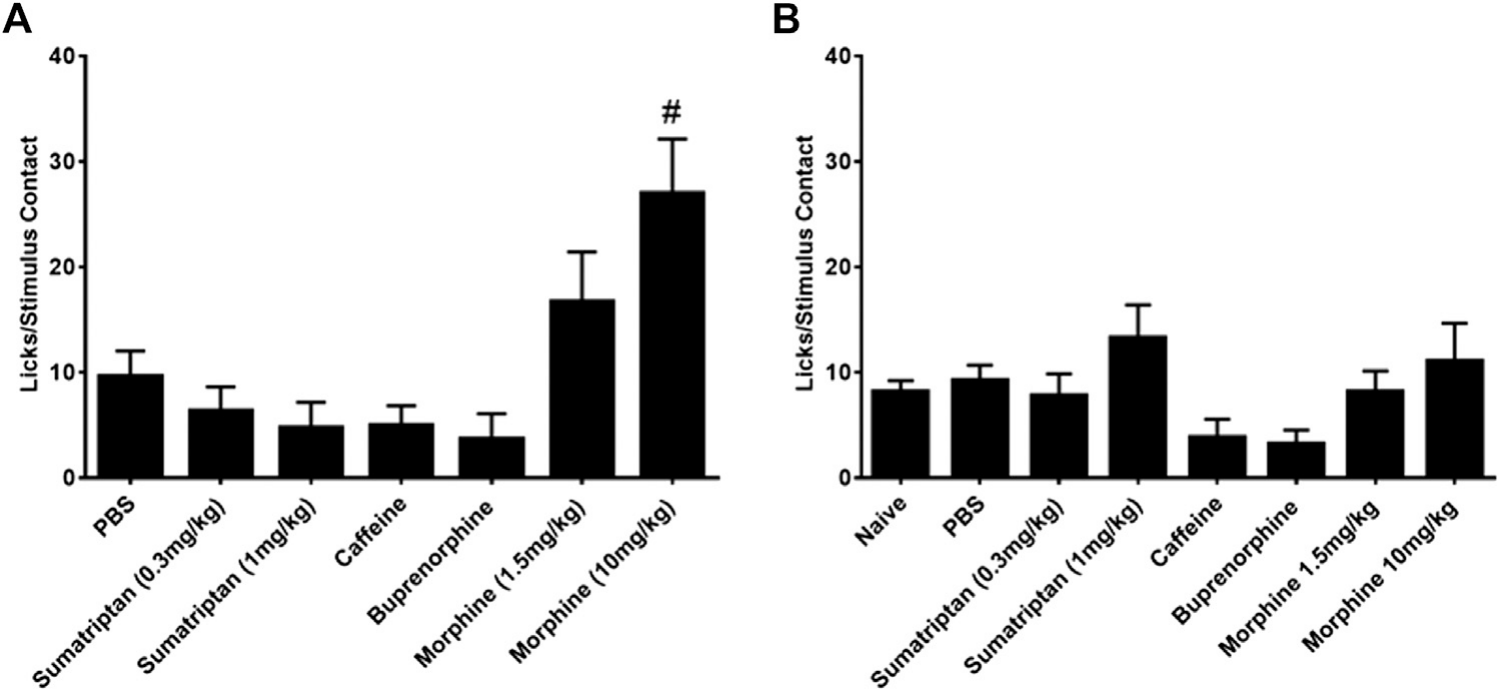
Influence of anti-nociceptive agents on the ratio of licks on the reward bottle to stimulus contacts. **(A)** Agents tested in the presence of 10 mg/kg nitroglycerin (i.p.). The low dose of sumatriptan (0.3 mg/kg) was given as a rescue. All other drugs were given preemptively. One-way ANOVA: F (6, 107) = 3.25, *p* = 0.0056, #*p* < 0.05 Dunnett’s test when compared to PBS. **(B)** Agents tested in the absence of nitroglycerin. One-way ANOVA: F (7, 150) = 1.55, *p* = 0.15.

**FIGURE 4:**
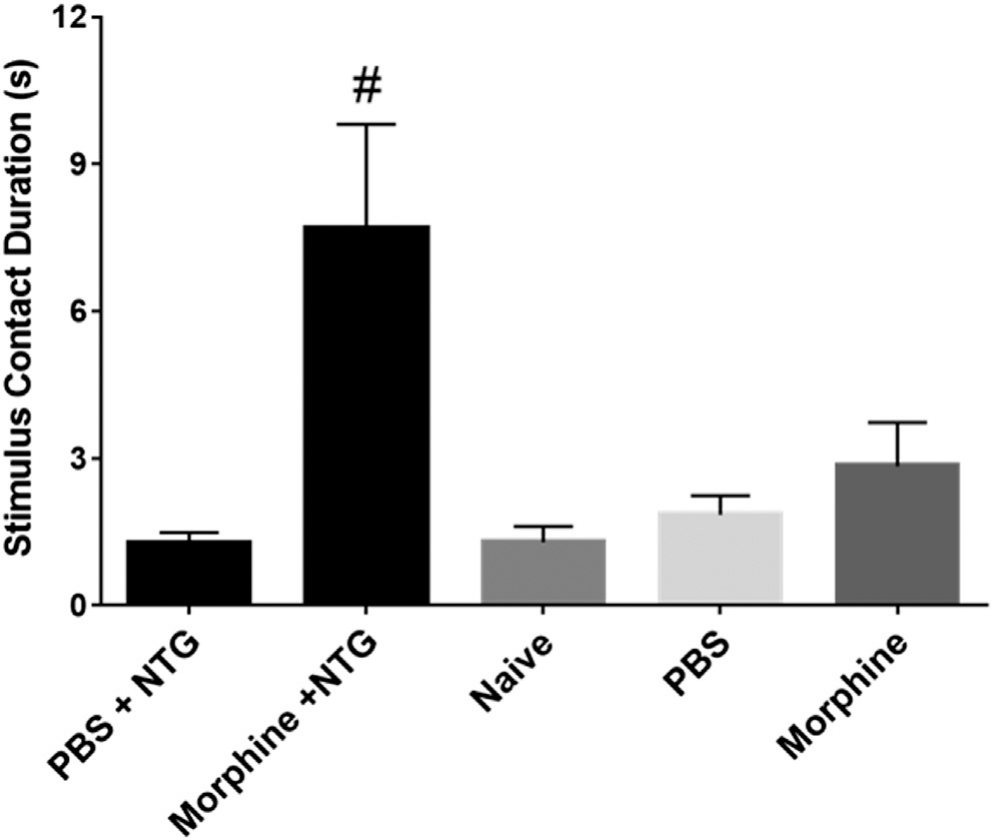
Effect of 10 mg/kg morphine (i.p.) and nitroglycerin on average stimulus contact duration in the OPAD assay (Mean ± SEM). One-way ANOVA: F (4, 45) = 6.297, *p* = 0.0004, #*p* < 0.05 Dunnett’s test when compared to PBS + NTG.

In contrast to the lack of effect of all the therapeutic agents on the number of licks and stimulus contacts, the combination of morphine and nitroglycerin produced a significant morphine dose dependent increase in the ratio of licks on the reward bottle to contacts with the stimulus bars (Figure 3A). No other agent produced this increase in the ratio. Morphine and the other agents had no effect on this ratio in the absence of nitroglycerin (Figure 3B). This finding was further evaluated by examining the average duration of the rodents’ contacts with the stimulus bars. The duration of the stimulus contacts in the presence of both nitroglycerin and 10 mg/kg morphine was significantly increased (Figure 4) which would provide more time for licking on the reward bottle during each bout. This finding suggests that although overall activity in the rats was reduced by the nitroglycerin treatment, the combination of nitroglycerin and morphine increased the rats’ tolerance of the mechanical stimulus when they attempted to obtain the reward solution.

### Effects of Nitroglycerin and Morphine on the Licking Motor Pattern Generator

**FIGURE 5:**
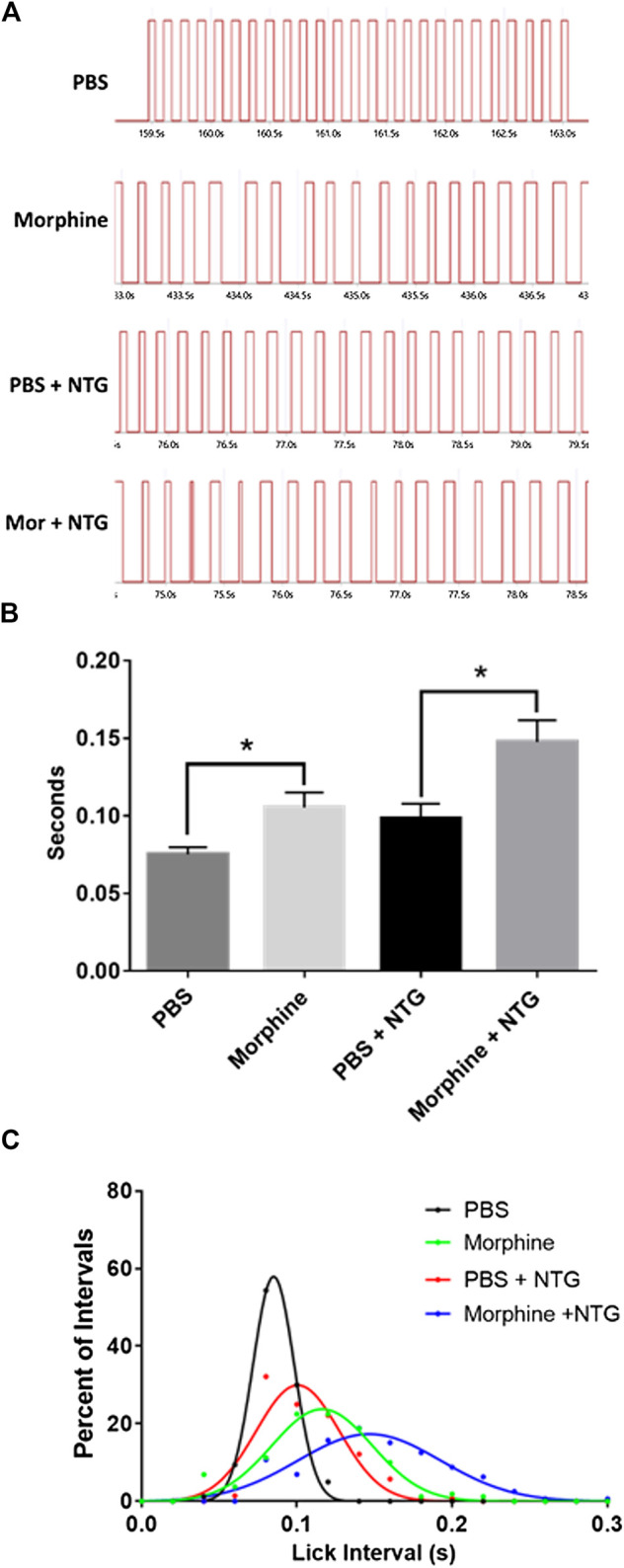
Nitroglycerin and morphine increase the interval between licks. **(A)** Representative licking bouts from animals treated with PBS, morphine (10 mg/kg), PBS + nitroglycerin (10 mg/kg), or morphine (10 mg/kg) + nitroglycerin (10 mg/kg) i.p. **(B)** Averaged interval between licks (Mean ± SEM). T-test PBS vs. morphine: *t* = 2.807 d*f* = 14, *p* = 0.014. T-test PBS + NTG vs morphine + NTG: *t* = 2.819 df = 13, *p* = 0.015. *N* = 7 to 8 rats per treatment group. **(C)** Frequency distribution of intervals between licks. The data demonstrate the increased variability of the intervals in the presence of morphine and/or nitroglycerin. Data was fitted with Gaussian curves using PRISM statistical software.

In analyzing the OPAD data it was observed that the frequency of the licking on the reward bottle was reduced by 10 mg/kg morphine and by nitroglycerin (Figure 5A). This was analyzed by averaging a sampling of 20 consecutive intervals between licks. As Figure 5B demonstrates both morphine and nitroglycerin significantly increase the lick interval and their effects appear to be additive when they are administered in combination. No other agents in the presence or absence of nitroglycerin produced a similar increase in the intervals between licks (Table 2). As illustrated by the frequency distribution of the intervals (Figure 5C), morphine, nitroglycerin and the combination of morphine and nitroglycerin also increased the variability of the intervals suggesting that these agents disrupt the rat tongue motor pattern generator.

**TABLE 2:** Effect of sumatriptan, caffeine, buprenorphine and low dose morphine on Lick interval.

Treatment	*N*	Lick interval (ms) Mean ± SEM	*p* Dunnett’s test vs. (PBS + NTG)
Nitroglycerin (10 mg/kg)			
PBS + NTG	7	98.1 ± 9.7	—
Sumatriptan 0.3 mg/kg	5	96.6 ± 9.2	>0.05
Sumatriptan 1.0 mg/kg	4	126.6 ± 9.7	>0.05
Caffeine	5	90.7 ± 7.1	>0.05
Buprenorphine	4	128.5 ± 7.2	>0.05
Morphine 1.5 mg/kg	9	109.3 ± 7.1	>0.05
No Nitroglycerin			
Naïve	19	71.9 ± 6.8	—
PBS	15	86.1 ± 3.7	—
Sumatriptan 0.3 mg/kg	6	89.1 ± 4.8	—
Sumatriptan 1.0 mg/kg	8	87.4 ± 6.5	—
Caffeine	6	88.6 ± 3.9	—
Buprenorphine	4	78.1 ± 8.4	—
Morphine 1.5 mg/kg	8	88.2 ± 5.3	—

Nitroglycerin: One-way ANOVA: F (5, 28) = 2.71, *p* = 0.04.

No Nitroglycerin: One-way ANOVA: F (6, 59) = 1.337, *p* = 0.26.

Animals that did not have 20 consecutive licks on the reward bottle were excluded from the analysis.

### Repeated Nitroglycerin Injections

**FIGURE 6:**
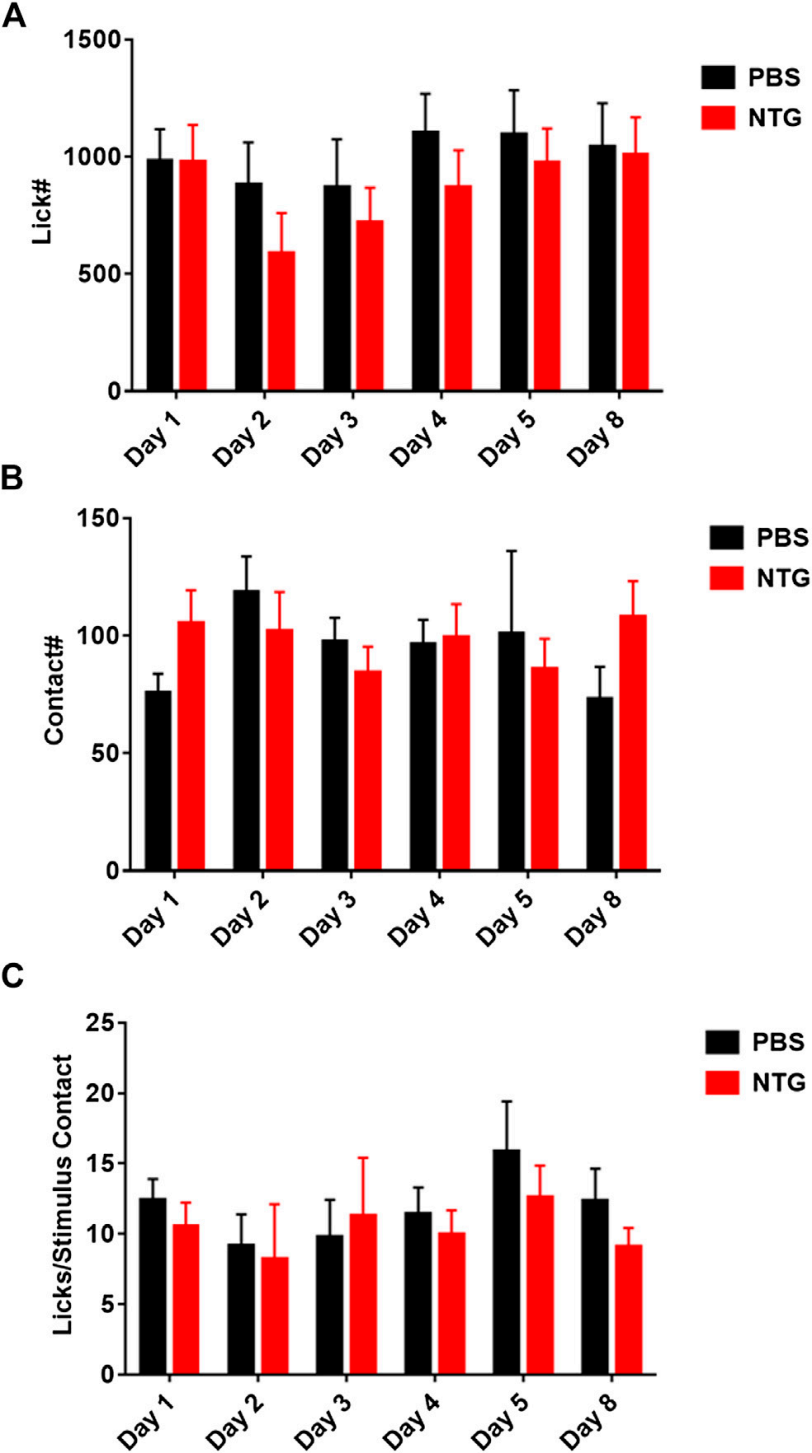
Repeated administration of nitroglycerin does not induce mechanical allodynia in the OPAD assay. Nitroglycerin (10 mg/kg i.p.) was administered on days 1–5 immediately following the rats’ session on the OPAD. **(A)** Effect of repeated nitroglycerin on licks on the reward bottle. One-way ANOVA: F (1, 28) = 0.4614, *p* = 0.50. **(B)** Effect of repeated nitroglycerin on the number of contacts with the mechanical stimulus. Two-way ANOVA: F (1, 28) = 0.07417, *p* = 0.79. **(C)** Effect of repeated nitroglycerin on the ratio of licks to stimulus contacts. Two-way ANOVA: F (1, 28) = 0.2838, *p* = 0.60. *N* = 15 rats per treatment group.

The overall reduction in motor activity produced by nitroglycerin suggested that the acute nociceptive, cardiovascular, or other effects of nitroglycerin may interfere with the assay and mask nitroglycerin induced hypersensitivity. Previous studies have reported that rodents develop allodynia when they receive multiple nitroglycerin injections over the course of several days (Farajdokht et al., 2018; Jeong et al., 2018). To determine if repeated injections of nitroglycerin produce hypersensitivity, we injected 10 mg/kg daily for five days immediately after testing in the OPAD. This design let the animals fully recover from the acute effects of nitroglycerin and allowed testing for the development of chronic or sustained allodynia. As Figure 6 illustrates, the treatments did not alter licking (6A), stimulus contacts (6B) or the ratio of licks to stimulus contacts (6C) indicating that chronic mechanical hypersensitivity was not produced by the treatment.

## Discussion

### Nitroglycerin in Female Rats Is Not a Model of Migraine Headache

In this reverse translation project, the rat nitroglycerin model of migraine headache was evaluated in an operant orofacial nociception assay. The hypothesis tested was that drugs that are used to reverse migraine headache would significantly reverse or prevent the effects of nitroglycerin in the rodent model. The OPAD assay evaluates nociception in regions innervated by trigeminal sensory neurons. The assay also collects all data electronically which significantly reduces investigator bias. Furthermore, OPADs utilize a pain suppressed behavior rather than pain enhanced behaviors like the von Frey mechanical assay. This prevents sedative or motor effects of a drug from being interpreted as anti-nociceptive. In the current study the data indicates that the nitroglycerin model in the current context does not meet the criteria that would indicate it is a model of human disease. 1) The mechanisms for nitroglycerin’s effects and for migraine headache are not established. Thus, it cannot be determined if nitroglycerin invokes the same mechanism of pathology as migraine. 2) The lack of trigeminal hypersensitivity suggests that the nitroglycerin is not producing the signs and symptoms of the disease. The one caveat to the assay is that the OPAD primarily measures nociception in the maxillary branch of the trigeminal nerve, whereas most hypersensitivity in human migraine is measured in the ophthalmic branch. However, many rodent studies report paw sensitivity following nitroglycerin injection, which is substantially more removed from the ophthalmic branch of the trigeminal nerve than the maxillary branch (Costa et al., 2005; Farkas et al., 2016; Farajdokht et al., 2018). 3) Although, the OPAD assay measures a clear and objective difference between the normal and disease state it is not evident that these measures are meaningful for the human disease. 4) The model did not reverse translate using effective therapies for the human disease as sumatriptan, morphine, buprenorphine, and caffeine did not impact the effects of nitroglycerin on nociceptive measures in the OPAD assay. Thus, we conclude that nitroglycerin in the current context is not a valid model of human migraine.

We have previously utilized the ratio of the licks on the reward bottle to the contacts with the stimulus as a “pain index” (Neubert et al., 2005; Neubert et al., 2006; Neubert et al., 2008; Nolan et al., 2011; Ramirez and Neubert, 2012; Rossi et al., 2012; Ramirez et al., 2015). This ratio decreases when the animals are not able to tolerate contact with the stimulus and increases when a drug suppresses the nociception produced by the stimulus. Thus, this ratio is indicative of the degree of hypersensitivity the animals experience. In this study the ratio was not altered by nitroglycerin until the maximum dose was tested. However, the highest dose of nitroglycerin reduced attempts to access the reward to an average of only 7.6 ± 2.6 events per session, which is too few to be confident of the validity of the ratio. Because lower doses of nitroglycerin did not alter the ratio it was concluded that nitroglycerin did not produce hypersensitivity in the face of the rats. If nitroglycerin produced trigeminal hypersensitivity its ED50 for hypersensitivity was substantially higher than for decreases in licks on the reward bottle and contacts with the stimulus. These finding are in contrast to previous publications which had indicated that acute administration of nitroglycerin produced hypersensitivity in the face (Di et al., 2016; Qin et al., 2018) and limbs (Tassorelli et al., 2003; Costa et al., 2005; Greco et al., 2008; Bobade et al., 2015) of rats. It should be noted that these studies used assays that required the investigator to interpret the response of the rodent. Since hypersensitivity in the face was reported in humans with migraine headaches an accurate rodent model of migraine headache should also produce allodynia in trigeminal nerve territories (Burstein et al., 2000a; Burstein et al., 2000b; Yarnitsky et al., 2003). Thus, nitroglycerin induced trigeminal hypersensitivity could not be verified in this study.

### Interaction Between Morphine and Nitroglycerin

Despite the model’s failure to imitate migraine headache there were a few interesting findings in this study that warrant further investigation. One observation was that the combination of morphine and nitroglycerin significantly increased the ratio of licks to stimulus contacts (Figure 3A). This was also evident by the increase in the duration of the stimulus contacts in the combination treated animals (Figure 4). Morphine did not produce this effect in the absence of nitroglycerin (Figures 3B and 4). This finding suggests that morphine and nitroglycerin work in concert to alter the rodent’s tolerance of the mechanical stimulus. This is particularly interesting because the overall number of licks on the reward and number of contacts with the stimulus were not significantly altered by the combination (Table 1). Yu Xu and colleagues previously demonstrated that nitric oxide can potentiate the opioid peptide ß-endorphin’s analgesic effects (Xu et al., 1995). Since nitroglycerin is a NO donor it is possible that the increase in the duration of the stimulus contact is due to mechanisms similar to those described by Yu Xu’s group. Interestingly, buprenorphine, a mixed opioid agonist/antagonist (Brasseur, 1997), did not demonstrate any interaction with nitroglycerin in our study. This finding suggests that nitroglycerin’s interaction with opioids may be limited to mu agonists.

Another interesting finding in our study was that both morphine and nitroglycerin increased the interval between licks, or decreased the frequency of licks, on the reward bottle to similar degrees. When the drugs were given together, they produced what appeared to be an additive effect (Figure 5).

Previous work has indicated that nitric oxide plays a role in slowing down motor central pattern generators by enhancing inhibitory neuronal circuitry (Foster et al., 2014) and mu agonists such as morphine are well known for their inhibition of respiratory central pattern generating neuronal circuits (Kokka et al., 1965; Levitt et al., 2015). Thus, the effects of nitroglycerin and morphine on licking behavior in this study are not entirely surprising. The licking central pattern generator is likely regulated by NO and mu opioids. The interaction of NO and opioids in this circuit deserves further study.

## Conclusion

Drug discovery is reliant on animal models of disease that ideally invoke the same pathological mechanisms as the human disease. It is also important that the model expresses the disease in an easily quantified manner.

In this project we reverse translated the rat nitroglycerin model of migraine headache to verify its validity. The results of our study indicate that this model is not suitable for studying migraine headache. Sumatriptan, morphine and caffeine, which are all used to treat migraine headaches did not alter the rats’ discomfort with the nitroglycerin treatment. However, there are a few caveats with our study. The study utilized only female rats because women suffer from migraine headaches more frequently than men (Stewart et al., 1994; Hu et al., 1999; Bonafede et al., 2017) and female rats are more sensitive to nitroglycerin than male rats (Supplementary Figure S1). The enhanced sensitivity of female rats was also previously reported by Greco et al. (2013). We also did not monitor estrous in this project since the variance in the dose response data was not significantly different than that found with male rats (Supplementary Figure S1). Other studies have used mice and male rats which may, for unknown reasons, be better suited models. The validity of mice and male rats in the OPAD nitroglycerin model remains to be determined. This project also utilized a behavioral assay that considers multiple features of the rodent’s experience, including motivation to access the reward solution. The therapeutic agents were clearly not able to block or reverse all the effects of nitroglycerin. But the agents may have had effects in the model that would be beneficial in treating migraine that the assay could not measure, such as a reduction in light sensitivity. Thus, further work is needed to completely rule out the use of nitroglycerin rodent models for migraine headache research.

## Data Availability Statement

All datasets generated for this study are included in the article/Supplementary Material.

## Ethics Statement

The animal study was reviewed and approved by University of Florida Institutional Animal Care and Use Committee.

## Author Contributions

SC and NF performed all experimental procedures. JN, ER, and RC designed the experiments, analyzed the data and interpreted the results.

## Funding

This project was funded by Velocity Laboratories, LLC, Alachua, Florida, United States. JN and RC are the founders of Velocity Laboratories, LLC.

## Conflict of Interest

JN and RC are co-founders of Velocity Laboratories, LLC, which provided funding for this project.

The remaining authors declare that the research was conducted in the absence of any commercial or financial relationships that could be construed as a potential conflict of interest.
