# Supplementary figures and images for: Pharmacological Characterization of Orofacial Nociception in Female Rats Following Nitroglycerin Administration
Source: Front Pharmacol. 2020 Dec 3;11:527495. doi: 10.3389/fphar.2020.527495 (PMC7744726; doi:10.3389/fphar.2020.527495)

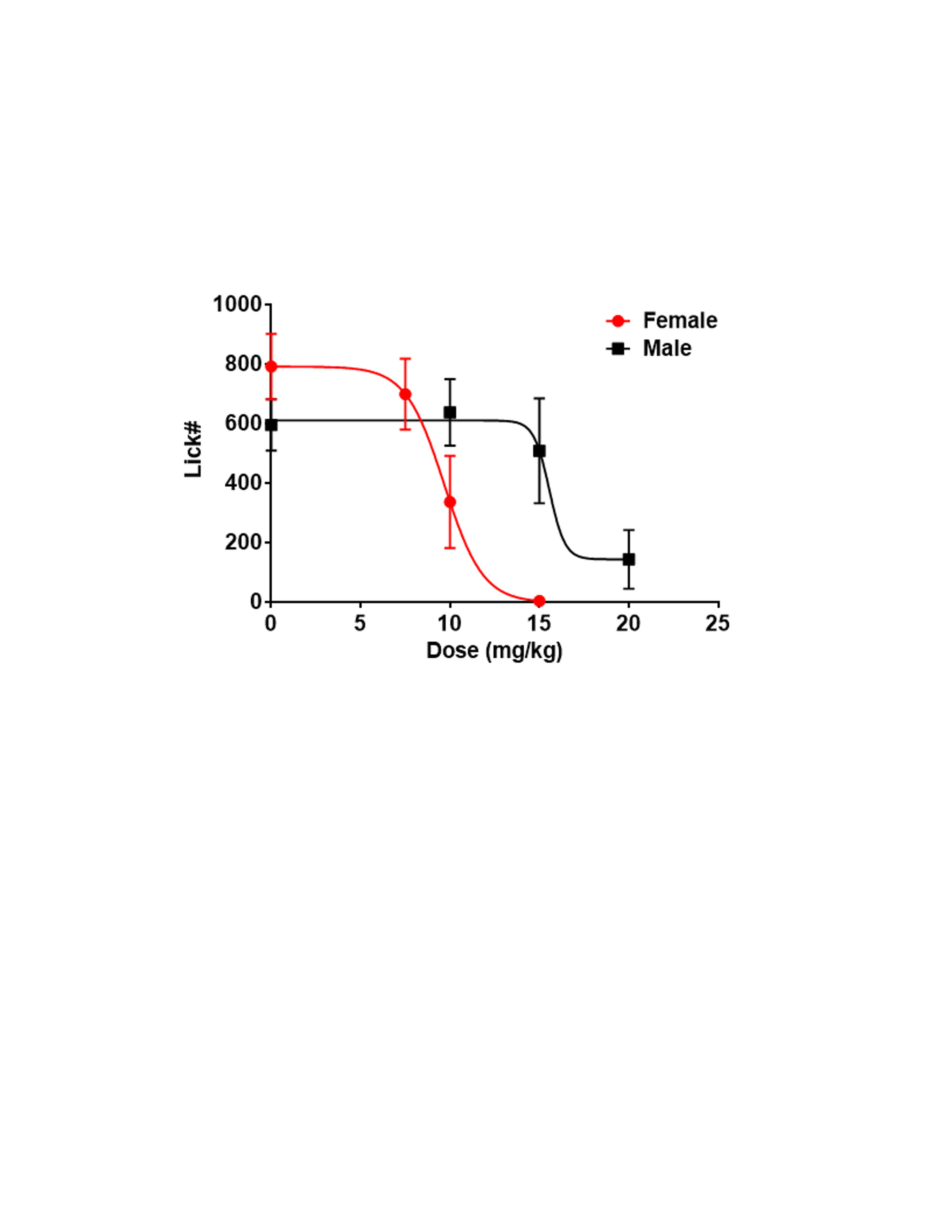

Supplement: Supplementary file 1 [file image1.tif]
